# Celecoxib and GABA Cooperatively Prevent the Progression of Pancreatic Cancer In Vitro and in Xenograft Models of Stress-Free and Stress-Exposed Mice

**DOI:** 10.1371/journal.pone.0043376

**Published:** 2012-08-16

**Authors:** Hussein A. N. Al-Wadei, Mohammed H. Al-Wadei, Mohammad F. Ullah, Hildegard M. Schuller

**Affiliations:** 1 Experimental Oncology Laboratory, Department of Biomedical and Diagnostic Sciences, College of Veterinary Medicine, University of Tennessee, Knoxville, Tennessee, United States of America; 2 Department of Preventive Medicine, Sana’a University, Sana’a, Yemen; Vanderbilt University Medical Center, United States of America

## Abstract

Pancreatic ductal adenocarcinoma (PDAC) has a poor prognosis and is associated with high levels of psychological distress. We have shown that beta-adrenergic receptors (β-ARs), which are activated by stress neurotransmitters, regulate PDAC cells via cyclic AMP (cAMP)-dependent signaling in vitro, that social stress promotes PDAC progression in mouse xenografts and that γ-aminobutyric acid (GABA) inhibits these responses in vitro and in vivo. The targeted inhibition of stress-induced regulatory pathways may abolish the potentially negative impact of psychological stress on clinical outcomes. Our current data show that chronic exposure of PDAC cell lines Panc-1 (activating point mutations in K-ras) and BXPC-3 (no mutations in K-ras) in vitro to the stress neurotransmitter epinephrine at the concentration (15 nM) previously measured in the serum of mice exposed to social stress significantly increased proliferation and migration. These responses were inhibited in a concentration-dependent manner by celecoxib. The effects of celecoxib alone and in combination with γ-aminobutyric acid (GABA) on the progression of subcutaneous mouse xenografts from the cell line (BXPC-3) most responsive to epinephrine were then investigated in the presence and absence of social stress. Cancer-stimulating factors (VEGF & prostaglandin E_2_ [PGE_2_]) and levels of cAMP were measured by immunoassays in blood and xenograft tissue. Phosphorylation of the signaling proteins ERK, CREB, Src, and AKT was assessed by ELISA assays and Western blotting. Expression of COX-2, 5-lipoxygenase, and p-5-LOX were determined by semi-quantitative Western blotting. Celecoxib alone significantly inhibited xenograft progression and decreased systemic and tumor VEGF, PGE2, and cAMP as well as phosphorylated signaling proteins in stress-exposed and stress-free mice. These responses were significantly enhanced by co-treatment with GABA. The celecoxib-induced downregulation of COX-2 protein and p-5-LOX was also significantly enhanced by GABA under both experimental conditions. Our findings identify the targeted inhibition of stress-induced pathways as a promising area for more effective cancer intervention in pancreatic cancer.

## Introduction

Cancer is responsible for approximately 13% of deaths worldwide and remains the second-leading cause of death in the United States, accounting for nearly one in every four deaths [1]. Statistically, pancreatic cancer is the fourth-leading cause of cancer-related deaths in developed countries and has a 5-year survival rate below 5% [2], [3]. It is one of the most deadly neoplastic diseases, as it is typically asymptomatic until it has reached an advanced stage when effective treatments are unavailing. At the time of diagnosis, most pancreatic cancers are therefore inoperable and have metastasized to distant organs. In addition, this malignancy is generally unresponsive to conventional radio-and chemotherapy, resulting in a mortality rate near 100% within 6 months of diagnosis [3]. Novel strategies for the improvement of pancreatic cancer intervention are therefore urgently needed.

Psychological stress as a potential modulator of cancer progression has recently emerged as an important new area of cancer research [4]–[6]. It has thus been shown that the growth and progression of the most common human cancers, including adenocarcinoma of the stomach [7], colon [8], prostate [9], mammary gland [10], [11], ovary [12], lung [13], [14] and pancreas [15], [16], are significantly stimulated by beta-adrenergic receptor (β-AR) signaling initiated by the stress neurotransmitters noradrenaline and adrenaline. It has also been shown that the epidermal growth factor receptor (EGFR) pathway is activated in pancreatic cancer cells by beta-adrenergic, PKA-dependent transactivation of the EGFR [17]. Additionally, β-ARs regulate the production of arachidonic acid (AA) in pancreatic cancer cells [15], resulting in the formation of cancer-stimulating AA metabolites. Recent investigations in pancreatic cancer xenografts from cell lines BxPC-3 and Panc-1 in mice exposed to social stress have further revealed significant stress-induced growth stimulation of the xenografts associated with the activation of multiple signaling proteins, including ERK, CREB, Src, and AKT [18], most of which are overexpressed in pancreatic cancer [19]–[21]. These findings suggest that psychological stress may decrease the efficacy of cancer intervention strategies. The targeted inhibition of stress-induced signaling cascades may therefore alleviate the negative impact of stress on clinical outcomes and also significantly reduce the incidence of pancreatic cancer developing in individuals at risk because of preexisting diabetes, pancreatitis, or chronic smoking [22].

The selective cyclooxygenase 2 (COX-2) inhibitor celecoxib blocks the formation of Cox-2-mediated, cancer-stimulating AA metabolites and has demonstrated promising preclinical and clinical anti-tumor activity in a variety of human tumors, including pancreatic cancer [23]–[25]. Celecoxib selectively inhibits COX-2 activity, which in turn regulates multiple pathways in pancreatic cancer [26]. Strong evidence additionally suggests that COX-2 also plays an important role in the development and progression of many non-pancreatic tumors [27]. COX-2 is highly expressed in a number of human cancers and cancer cell lines, including colon [28], gastric [29], squamous carcinoma of the head and neck [30], cervical [31], non-small cell lung [32], breast [33], prostate [34], and pancreatic cancer [35]. On the other hand, gamma-amino butyric acid (GABA) acts as the physiological inhibitor of the β-adrenergic cascade, an effect transduced intracellularly in pancreatic cancer cells by the Gα_i_-coupled serpentine GABA-B receptor that decreases the formation of cyclic AMP (cAMP) by inhibiting adenylyl cyclase activation [18], [36].

In the current study, we have investigated the effects of chronic exposure to the stress neurotransmitter epinephrine at a concentration (15 nM) previously measured by us in the serum of mice exposed to social stress [18] on the proliferation and migration of PDAC cell lines Panc-1 and BXPC-3 in vitro and explored potential inhibitory effects of celecoxib on these responses. We then used an established method for the experimental induction of chronic social stress, the predominant form of psychological stress in people [37], in mice carrying xenografts from the cell line (BXPC-3) which was most responsive to epinephrine in vitro, to investigate the potential inhibitory effects of celecoxib and GABA alone and in combination on xenograft progression in stress-free and stress-exposed animals. Celecoxib alone demonstrated strong cancer-inhibiting effects under both of these experimental conditions while the combination treatment with GABA significantly improved all investigated preventive outcomes. This study has implications for cancer progression and prevention in populations under chronic psychological distress due to socio-economic and personal challenges, which are acerbated further when a person is diagnosed with a life-threatening disease such as pancreatic cancer.

## Materials and Methods

### Culture of Cancer Cell Lines

The authenticated (RADIL, Columbia, MO), human pancreatic cancer cell lines BXPC-3 (witout ras mutations) and Panc-1 (expresses activating point mutations in K-ras) originally purchased from the American Type Culture Collection (Rockville, MD) were grown in RPMI-1640 with 10% fetal bovine serum without antibiotics.

### In Vitro Investigations

#### A) Determination of cell proliferation by MTT assay

The colorimetric 3-(4, 5-dimethyle thiazol-2-yl)-2, 5-diphenyl tetrazolium bromide (MTT) assay (Sigma) was used as previously described [38] to assess the growth stimulating effects of chronic epinephrine exposure and potential inhibitory effects of celecoxib on this response in Panc-1 and BXPC-3 cells in vitro. Briefly, Panc-1 and BxPC-3 cells were seeded into 6-well plates (50,000 cells per well). Cells were then pretreated with 15 nM epinephrine for 7 days (medium containing epinephrine was replaced every 24 hours) or cultured for 7 days without epinephrine. Celecoxib (1 nM to 100 µM) was then added to cells from both treatment groups. Following a 72 hour incubation period, all cells were harvested.

#### B) Assessment of cell migration by colorimetric assay

Cell migration assays were conducted as previously described [36], using 6-well plates with filter inserts provided by the colorimetric cell migration assay kit (Cell Biolabs, San Diego, CA, USA). Panc-1 and BxPC-3 cells were pretreated with 15 nM epinephrine for 7 days (medium containing epinephrine was replaced every 24 hours) in tissue culture flasks or cultured without epinephrine for 7 days. Cells from both treatment groups were then seeded into the inserts and treated with celecoxib (1 nM to 100 µM) for 24 hours. The migratory ability of cells was then assessed following the vendor’s instructions.

#### C) Statistical evaluation of in vitro data

**Figure 1:**
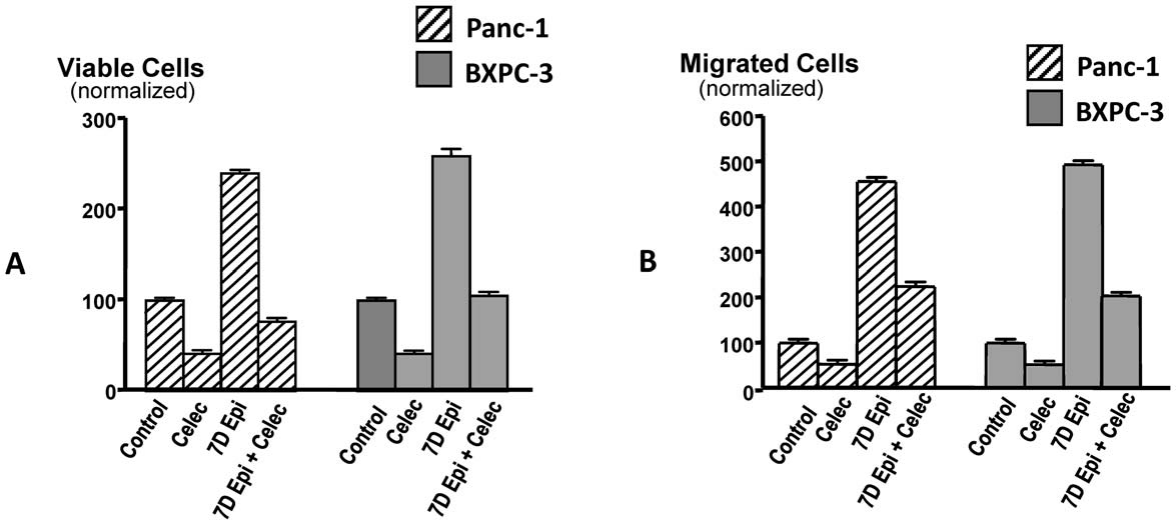
Results of MTT and migration assays. MTT assays (A) in Panc-1 (with activating point mutations in K-ras) and BXPC-3 (without ras mutations) cells in vitro. Exposure for 7 days to epinephrine (15 nM) significantly (*p*<0.001) increased the number of viable cell in both cell lines. The Cox-2 inhibitor celecoxib (1 µM) completely blocked this response to epinephrine (*p*<0.001) while additionally reducing (*p*<0.001) the number of viable cells in cells not pre-treated with epinephrine. The effects of identical treatments on cell migration are shown in Fig. B. In both cell lines migration was significantly more stimulated by chronic epinephrine than cell proliferation. Celecoxib significantly (*p*<0.001) reduced epinephrine-induced migration but did not completely block this response. BCPC-3 cells were slightly more responsive to epinephrine in both assays, but the differences between the two cell lines were not significant. Columns are mean values and standard deviations of 5 samples per treatment group.

**Figure 2:**
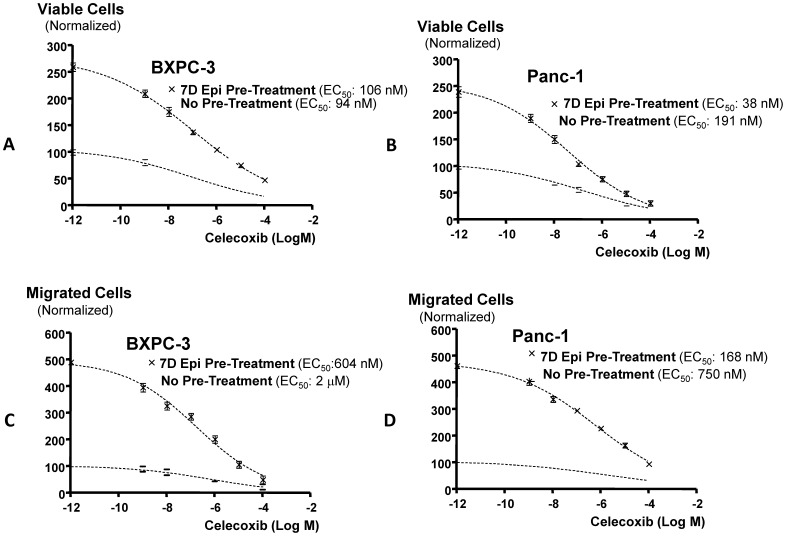
Cell proliferation and migration dose response-curves. Dose response-curves for celecoxib in Panc-1 and BXPC-3 cells in vitro in the presence and absence of pretreatment for 7 days with epinephrine (15 nM). Figures A and B show cell proliferation responses in MTT assays while Figures C and D show cell migration responses. While both cellular responses were similar among the two cell lines, EC_50_ values for celecoxib in Figs. B, C and D were significantly (*p*<0.001) lower in the cells pretreated for 7 days with epinephrine. Data points are mean values and standard deviations (n = 5). EC_50_ values and curve fit were established by nonlinear regression analysis for sigmoidal dose-responses.

Data (n = 5) in column graphs (Fig. 1) from the cell proliferation and cell migration assays in the presence and absence of chronic epinephrine treatment were assessed by one-way analysis of variance followed by Dunn’s multiple comparison test. Data (n = 5) from the dose-response experiments (Fig. 2) with celecoxib were fitted to sigmoidal dose-response curves and EC_50_ values for celecoxib were calculated by nonlinear regression analysis using Prism GraphPad software.

### In Vivo Investigations

#### A) Animal experiment

Six-week-old male athymic nude mice were purchased from Harlan Sprague Dawley. The animal research protocol was approved by the University of Tennessee Institutional Animal Care and Use Committee. The mice were acclimated for 1 week and maintained in our laboratory animal facility in accordance with guidelines of the American Association of Laboratory Animal Care under standard laboratory conditions in which temperature, humidity, and light are controlled, and had free access to autoclaved Purina Rodent Chow food and autoclaved water. Following the 1-week acclimation, the ears of the mice were tagged to facilitate the monitoring of each animal, which was then randomly assigned to six treatment groups (*n* = 20), with 5 mice per cage. Before tumor cell inoculation, mice in the three psychological stress groups were exposed to social stress for 4 weeks according to the published procedure [37] by changing the group composition of each cage twice a week. BXPC-3 cells that had reached 75% confluency in culture were then subcutaneously inoculated in the flank region (3×10^6^ in 0.2 ml of PBS, viability >95%) of animals from each of these groups. Social stress was continued in these three groups for another 30 days. Three other groups of mice that were not exposed to social stress were also inoculated with identical numbers of pancreatic cancer BXPC-3 cells. One group each from the social stress and non-social stress populations was treated by intraperitoneal injections of celecoxib (Celebrex, Pfizer; 25 mg/Kg b.w., 5 days/week for 30 days). Additionally, one group each from the social stress and non-social stress populations was simultaneously treated by initial intraperitoneal injections of celecoxib (25 mg/Kg b.w., 5 days/week for 30 days) and then GABA (Sigma; 10 mg/Kg b.w., 5 days/week for 30 days) immediately thereafter on the mouse’s opposite side. All animals were observed for 30 days after inoculation with cancer cells. Tumor sizes were evaluated weekly by digital caliper, and two perpendicular diameters (length and width) of each xenograft were measured using the following formula: tumor volume = (length/2)×(width^2^). The weight of the animals was followed throughout the experiment to monitor their general health state and for GABA and celecoxib treatment effects. At the end of the 30-day observation period, the animals were euthanized by CO_2_ inhalation. Blood samples were collected for the determination of neurotransmitters, vascular endothelial growth factor (VEGF), and prostaglandin E_2_ (PGE_2_) in the serum and of cAMP in the cellular fraction of blood. The tumors were excised, snap frozen in liquid nitrogen, and then stored at −80°C for further analyses.

#### B) Immunoassays for the quantitative analysis of the second messenger cAMP, VEGF, PGE2, and phosphorylated signaling proteins

VEGF and PGE_2_ in serum and tumor tissue samples and cAMP in the cellular fraction of blood and tumor tissues samples were analyzed by immunoassays as recommended by the manufacturer (Enzo Life Sciences International). Briefly, the kit for measurement of cAMP uses a polyclonal antibody to bind a competitive manner the cAMP in the standards and samples. The kit for measurement of VEGF uses monoclonal antibody to human VEGF immobilized in a microtiter plate to bind the VEGF in the standards or samples. The PGE_2_ kit uses a monoclonal antibody to PGE_2_ to bind in a competitive manner the PGE_2_ in the samples and standards. Absorbances were read with an ELISA reader at 405 nm for cAMP, 450 nm for PGE_2_, and at 450 nm for VEGF (*n* = 5 per treatment group). Detection and quantitation of levels of p-ERK-1/2 at threonine 202 and tyrosine 204, p-CREB at serine at 133, and p-Akt at serine residue 473 (Invitrogen); and kinase activity of the recombinant catalytic domain of p-Src (MBL International) were conducted from tumor tissue samples (*n* = 5 per treatment group) homogenized in RIPA buffer and protease inhibitors as recommended by the manufacturers. Absorbance was read with an ELISA reader at 450 nm. Serum and tumor levels of the stress neurotransmitters noradrenaline and adrenaline as well as GABA were determined by ELISA assays as previously described [14], [18] to monitor the successful induction of psychological stress (data not shown).

#### C) Semi-quantitative analysis of COX-2, 5-lipooxygenase (5-LOX) and p-5-LOX, and non-quantitative visualization of signaling proteins by western blotting

Three independent Western blots were conducted for each antibody for the semi-quantitative assessment of protein expression by densitometry for the AA-metabolizing enzymes COX-2 and p-5-LOX, using NIH Image J software. In addition, protein expression of the signaling molecules quantitated by ELISA assays above were visualized by non-quantitative Western blots. Briefly, tumor tissues were briefly homogenized in RIPA lysis buffer (Thermo Scientific), PMSF, Na_3_VO_4_, and 1 mg/ml aprotinin, leupeptin, and pepstatin. After heat denaturation at 100°C for 5 min, equal amounts of protein were electrophoresed using 12% Novex SDS-polyacrylamide gels (Invitrogen) and transferred onto nitrocellulose membranes; Western blots were then performed using incubation overnight at 4°C with the following primary antibodies: total ERK1/2, p-ERK1/2, p-CREB, AKT p-AKT, Src, p-Src, COX-2, 5-LOX, and p-5-LOX (Cell Signaling). Total CREB and actin were purchased from Millipore. Secondary antibodies were incubated for 1 h at room temperature. Bands were visualized by ECL (Pierce, Thermo Scientific).

#### D) Statistical analysis of in vivo data

Statistical analysis was performed using GraphPad Instat software (GraphPad Software Inc., La Jolla, CA, USA). To test if the variation among column medians of tumor volumes in the six treatment groups (*n* = 20) was significantly greater than expected by chance, nonparametric Kruskal-Wallis ANOVA was performed for data from each of the 4 weeks after injection of tumor cells. Differences between selected pairs of treatment groups were additionally assessed by the nonparametric Mann-Whitney test. Statistical significance of differences among groups for levels of cAMP (*n = *5), PGE_2_ (*n = *5), and VEGF (*n* = 5) in blood and xenograft tissues and for p-ERK, p-CREB, p-Src, and p-AKT in tumor tissue (*n* = 5 per treatment group) was assessed by the non-parametric Mann-Whitney test. Statistical significance of differences between four densitometric readings per protein band from three independent Western blots for the semi-quantitative assessment of COX-2 and p-5-LOX prepared from three randomly selected xenografts per treatment group (*n* = 12) was assessed by the non-parametric Mann-Whitney test.

## Results

### Chronic Exposure to Epinephrine Increases, and Celecoxib Inhibits Cell Proliferation and Migration

Chronic exposure of Panc-1 and BxPC-3 cells to epinephrine significantly (*p*<0.0001) increased cell proliferation and migration, with BXPC-3 cells being more responsive to epinephrine in both assays (Fig. 1). Treatment of the cells with celecoxib (1 µM) completely abolished these responses in both cell lines (*p*<0.001) (Fig. 1). Celecoxib (1 µM) also significantly (*p*<0.001) inhibited cell proliferation and migration in unpretraeted cells from both cell lines (Fig. 1). As shown in the dose-response curves (Fig. 2), celecoxib inhibited cell proliferation and migration in both cell lines in the presence and absence of chronic epinephrine pre0treatment in a concentration dependent manner. The numbers of viable and migrated cells in epinephrine pretreated cells from both cell lines were significantly (*p*<0.001) greater than in unpretreated cells at all celecoxib concentrations tested, illustrating the strong PDAC stimulating effects of epinephrine (Fig. 2). At the same time, EC_50_ values for celecoxib were significantly (*p*<0.001) lower in epinephrine pretreated cells from both cell lines than in the unpretreated cells (Fig. 2), suggesting strong dependence of epinephrine-induced migration on COX-2 mediated arachidonic acid metabolites.

### Modulation of Xenograft Volumes by Celecoxib and GABA in Stress-exposed and Unstressed Mice

**Figure 3:**
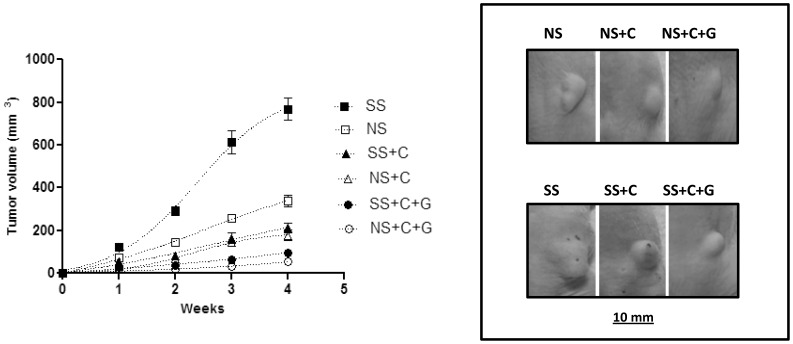
Mean values and SE of xenograft volumes over time from the BXPC-3 cell line in groups of mice (*n* = 20). Animals were exposed to social stress (SS) or not exposed to stress (NS) in the presence and absence of celecoxib (C) or celecoxib and GABA (C+G) treatment. The xenograft volume was significantly increased (*p*<0.0001) by social stress. This response was significantly inhibited by treatment with celecoxib alone (*p*<0.001) or by celecoxib and GABA (*p*<0.0001). Overall, the differences in tumor volumes were statistical significant (*p*<0.001) between treated and untreated groups. Photographs show representative examples of xenografts for each treatment group.

Statistical analysis of data from all six treatment groups by nonparametric ANOVA yielded variations among column means significantly (*p*<0.0001) greater than expected by chance in each of the observed 4 weeks. As previously reported [18], the progression of xenograft growth from the BxPC-3 pancreatic cancer cell line was significantly (week 1: *p*<0.006; weeks 2–4: *p*<0.0001 by Mann-Whitney test) promoted by psychological stress throughout the observation period of 4 weeks (Fig. 3). Treatment of unstressed animals with celecoxib alone significantly (*p*<0.0001) reduced xenograft sizes at all time intervals measured. In addition, celecoxib alone reduced xenograft sizes in stress-exposed mice below the levels of untreated, stress-free animals (*p*<0.001 in each of the 4 observed weeks). Treatment of unstressed mice with celecoxib in combination with GABA further enhanced the growth-inhibiting effects of celecoxib (week 1: *p*<0.0376; weeks 2–4: *p*<0.0001). Similarly, co-treatment with GABA also increased the cancer-inhibiting effects of celecoxib in the stress-exposed group (*p*<0.0001 in weeks 1–4).

### Modulation of cAMP and PGE2 Levels in Serum and Tumor Tissue by Celecoxib and GABA in Stress-exposed and Unstressed Mice

**Figure 4:**
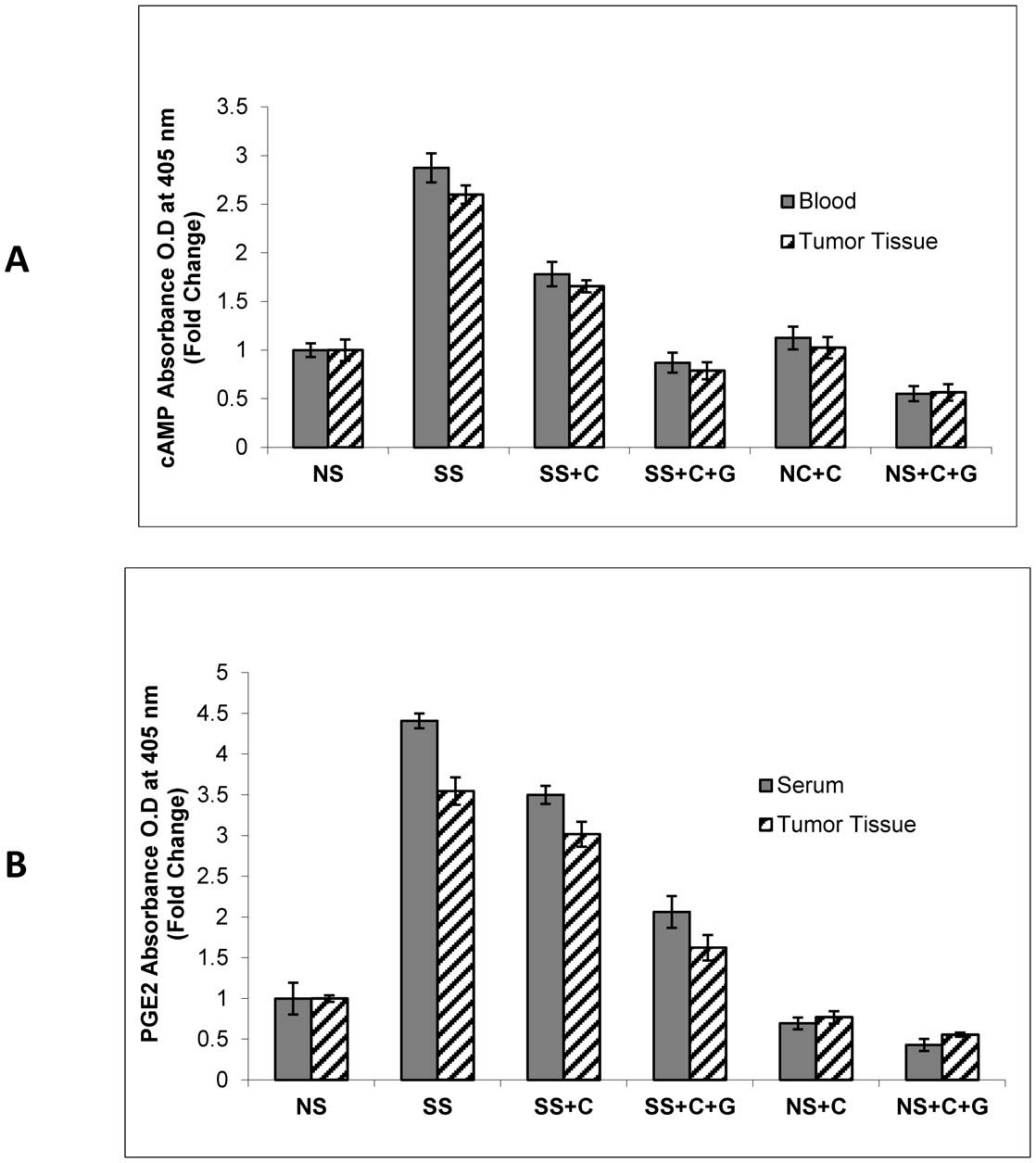
ELISA immunoassays demonstrating levels of intracellular cAMP and PGE2. cAMP was assessed in the cellular fraction of blood and in xenograft tissues (A) and levels of PGE_2_ in serum and xenograft tissues (B). Social stress increased the levels of both factors significantly (*p*<0.001), responses significantly (*p*<0.001) reduced by celecoxib. Combined celecoxib/GABA significantly (*p*<0.001) enhanced the inhibitory effects of celecoxib (*p*<0.001) on systemic and tumor levels of cAMP and PGE2 in stress free and social stress-exposed mice. Columns are mean values and SD of 5 randomly-selected samples per treatment group.

Beta-adrenergic agonists activate the production of AA in pancreatic cancer cells, leading to the COX-2-mediated formation of PGE2 [15]. Beta-adrenergic receptors [39] as well as PGE2 receptors [40] are coupled to the stimulatory G-protein Gαs that increases cAMP levels via activation of adenylyl cyclase. We therefore measured cAMP in the cellular fraction of blood and tumor tissue and PGE2 in serum and tumor tissues by ELISA assays. Our data show that the systemic levels of cAMP in blood samples and tumor tissues were significantly (*p*<0.001) increased (2.8-fold in blood and 2.5-fold in tumor tissues) in the mice exposed to stress. This stress response in blood cells and tumors was significantly (*p*<0.001) reduced by treatment with celecoxib alone, an effect further enhanced (*p*<0.001) by the combination with GABA (Fig. 4A). The observed levels of PGE2 in serum and tumor tissues followed a similar pattern, with significant (*p*<0.0001) increases in the stress-exposed animals, responses significantly (*p*<0.001) reduced by celecoxib alone and further decreased by the celecoxib/GABA combination (Fig. 4B). Combined celecoxib/GABA treatment additionally significantly (*p*<0.001) reduced systemic and tumor levels of cAMP and PGE2 in unstressed mice (Fig. 4A, 4B).

### Downregulation of Phosphorylated Signaling Proteins in Tumor Tissues by Celecoxib and GABA in Stress-exposed and Unstressed Mice

**Figure 5:**
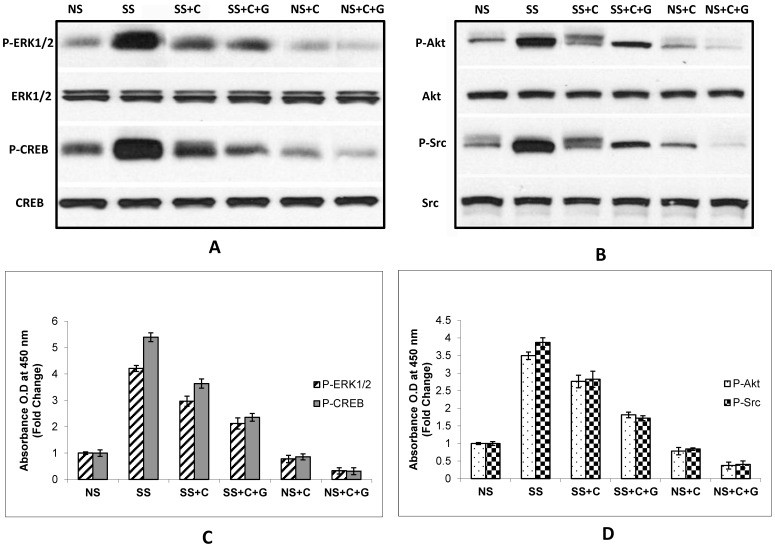
Protein expression of p-ERK and p-CREB (A) or p-AKT and p-Src (B) in BxPC-3 xenografts of stress free (NS) and social stress (SS) exposed mice. Western blots (A and B) illustrating induction of p-ERK, p-CREB, p-AKT and p-Src by social stress and inhibition of these responses by celcoxib alone or celecoxib + GABA. Quantitative assessment of these changes at the protein level were determined in the ELISA assays shown in Figure C and D. Induction of all investigated phosphorylated proteins by social stress (SS) was significant (*p*<0.001). Inhibition of these responses by celcoxib alone or the combination of celecoxib + GABA were significant (*p*<0.001) for all phosphorylated proteins. Columns in the graphs (C and D) are mean values and SD of quantitative ELISA immunoassays from five randomly-selected xenografts per treatment group.

It has been previously shown that β-adrenergic signaling phosphorylates multiple signaling proteins in human pancreatic cancer cells in vitro, including CREB, ERK, Akt, and Src [17], [38] and that the phosphorylated forms of these proteins are also induced in pancreatic cancer xenografts of stress-exposed mice [18]. We therefore visualized the expression of the phosphorylated and unphosphorylated forms of these proteins in tumor tissues by Western blotting and determined quantitative changes in their phosphorylation levels by ELISA assays. The stress-induced (*p*<0.0001) phosphorylation of each of these investigated signaling proteins was significantly (*p*<0.0001) reduced by treatment with celcoxib alone, an effect significantly (*p*<0.0001) increased by the additional treatment with GABA (Fig. 5 A–D). In addition, combined treatment with celecoxib and GABA significantly (*p*<0.0001) decreased the expression of the phosphorylated forms of all investigated signaling proteins in mice not exposed to stress (Fig. 5 A–D).

### Modulation of COX-2 and 5-LOX in Tumor Tissue by Celecoxib and GABA in Stress-exposed and Unstressed Mice

**Figure 6:**
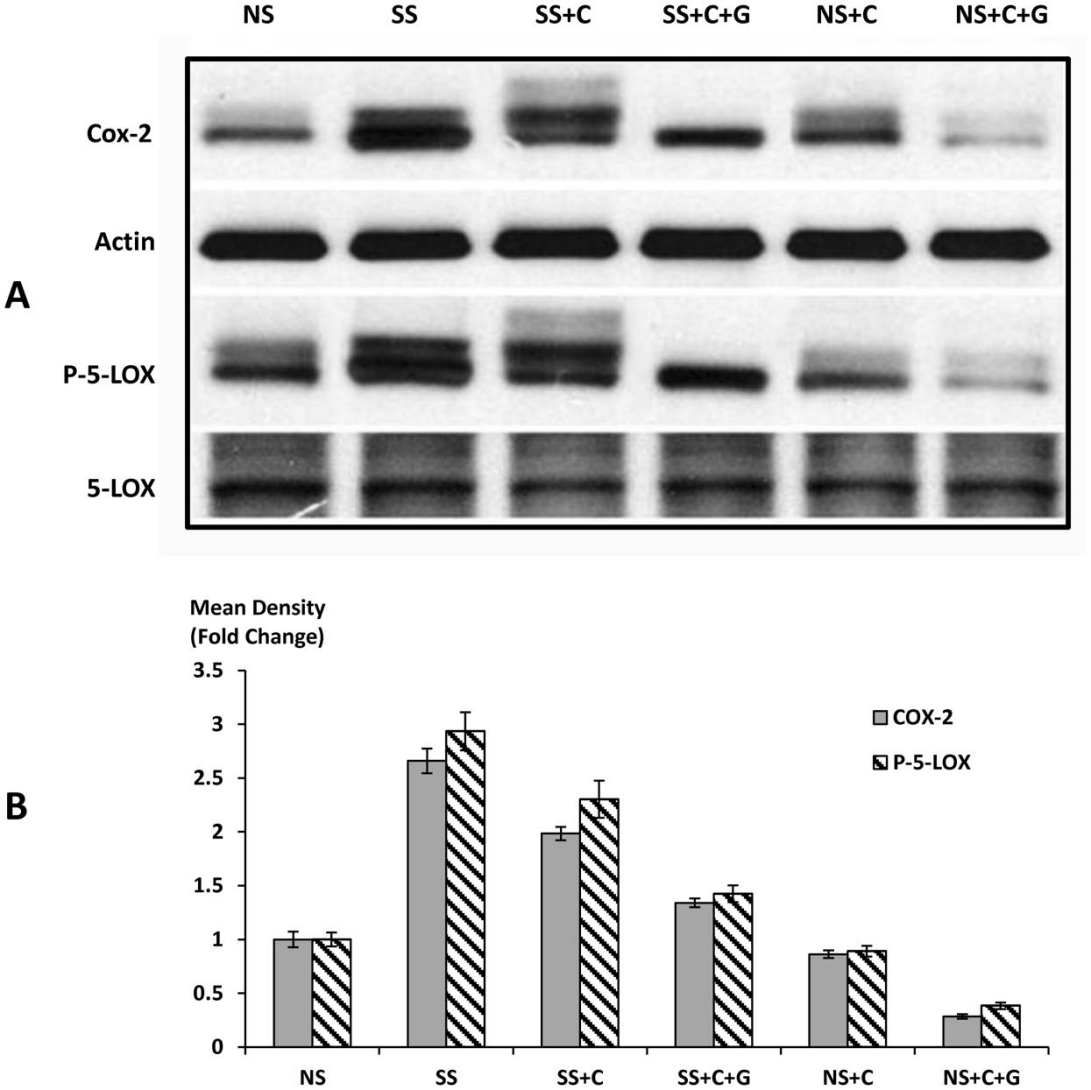
Protein expression of COX-2 and p-5-LOX. Western blot (A) is representative of three independent blots prepared from three randomly-selected xenografts per treatment group. Semi-quantitative densitometry of the bands from three independent Western blots showed significant (*p*<0.0001) increases in protein expression by social stress. These responses in turn were significantly reduced by celecoxib treatment alone (*p*<0.001) or by celecoxib and GABA (*p*<0.0001). Columns in the graph (B) are mean values and SD of four densitometric readings per band adjusted for actin in independent blots prepared from three randomly-selected xenografts per treatment group.

The enzyme COX-2 catalyzes the formation of prostaglandins such as PGE2 from AA while 5-LOX mediates the metabolic conversion of AA to leukotrienes [41]. Both enzymes are frequently overexpressed in pancreatic cancer [42], and the phosphorylation of 5-LOX at Ser-271 is promoted by AA, a process catalyzed by several kinases, including PKA [43]. We therefore investigated the expression levels of COX-2, 5-LOX, and p-5-LOX by semiquantitative Western analysis in the six treatment groups of our experiment. Our data show that stress significantly (*p*<0.0001) induced the expression of COX-2 and p-5-LOX (2.7 fold and 2.9 fold, respectively; Fig. 6A, 6B). Celecoxib alone significantly (*p*<0.001) reduced the stress-induced upregulation of COX-2, an effect significantly (*p*<0.001) enhanced by the added treatment with GABA (Fig. 6A, 6B). In addition, the combined treatment with celecoxib and GABA significantly (*p*<0.0001) downregulated the expression of COX-2 (0.4 fold) and p-5-LOX (0.5 fold) in mice not exposed to stress.

### Modulation of VEGF Levels in Serum and Tumor Tissue by Celecoxib and GABA in Stress-exposed and Unstressed Mice

**Figure 7:**
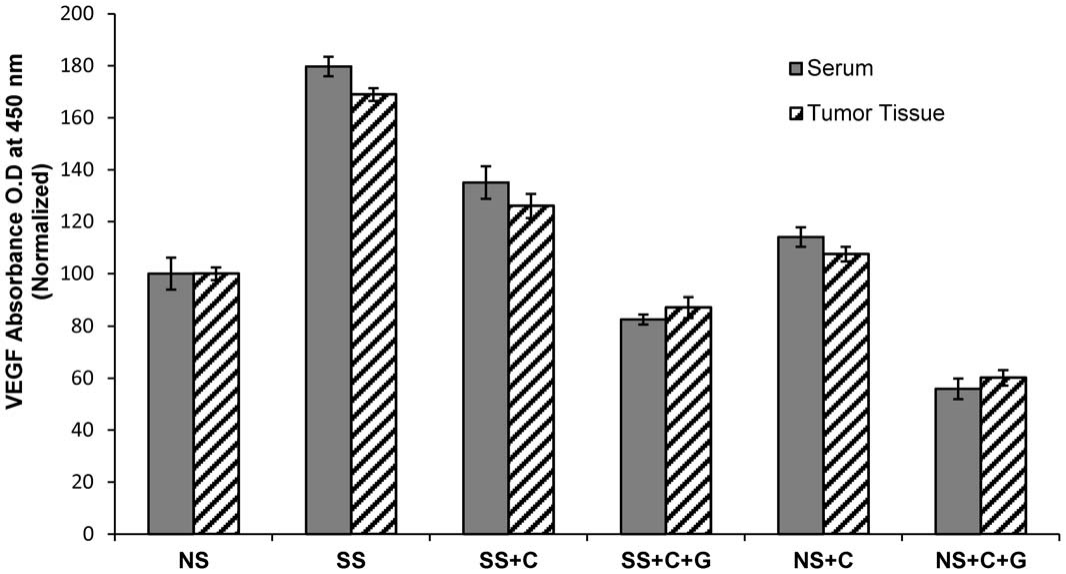
ELISA immunoassays demonstrating levels of VEGF in serum and xenograft tissues. Social stress significantly (*p*<0.001) increased VEGF levels in both serum and xenografts, a response significantly reduced by celecoxib (C, *p*<0.001) while celecoxib and GABA (C+G) treatment completely blocked the stress-induced induction of VEGF in serum and tumor tissue. In addition, celecoxib plus GABA significantly reduced VEGF below basal levels in mice not exposed to stress (*p*<0.001). Columns are mean values and SD of five randomly-selected samples per treatment group.

VEGF is the most potent and specific angiogenic growth factor [44]. Social stress significantly (*p*<0.001) increased VEGF levels in both serum (1.8 fold) and xenografts (1.7 fold) of mice carrying xenografts from the BxPC-3 cell line. Celecoxib alone significantly (*p*<0.001) reduced both responses while combination treatment with celecoxib/GABA completely blocked the stress-induced induction of VEGF in serum and tumor tissue (Fig. 7). In addition, celecoxib plus GABA significantly (*p*<0.001) reduced VEGF below base levels (*p*<0.001) in unstressed mice (Fig. 7).

## Discussion

**Figure 8:**
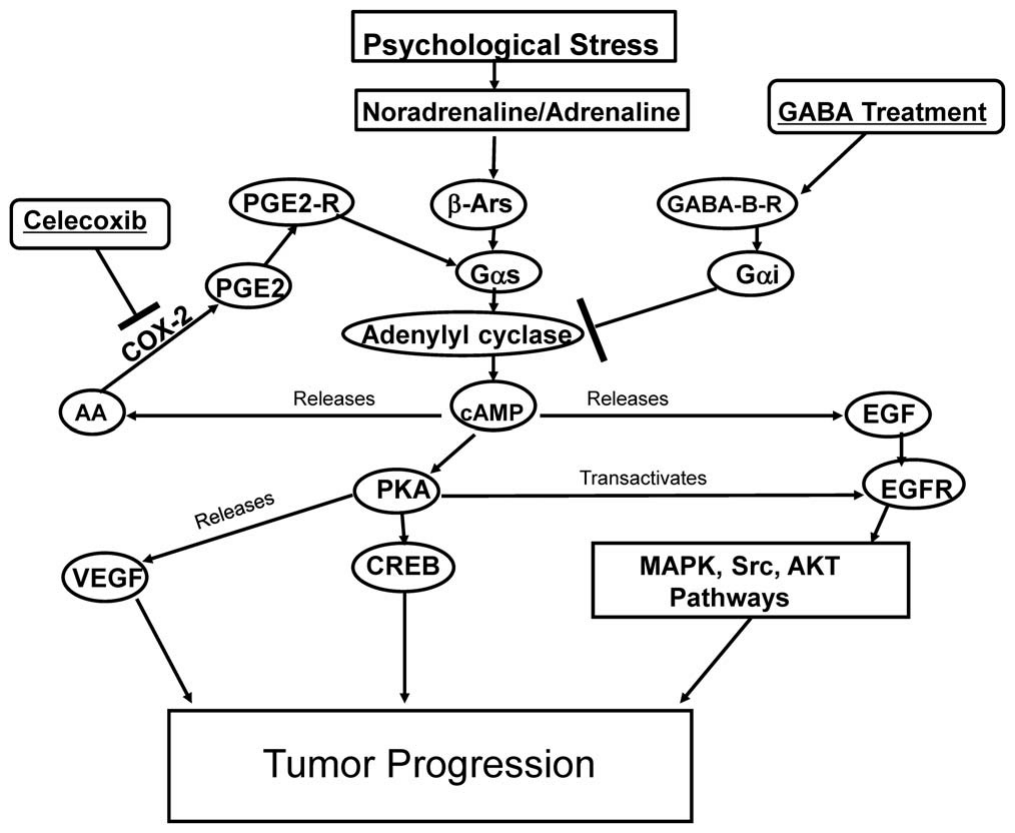
Simplified working model showing the effects of celecoxib and GABA. Xenograft growth from human pancreatic cancer cell line BxPC-3 was induced in mice by psychological stress via the interaction of β-adrenergic and COX-2 signaling pathways. Adenylyl cyclase activation downstream of the β-ARs induces the cAMP-PKA-CREB pathway, transactivates EGFR, and induces the release of EGF and AA, which in turn enhances VEGF and PGE_2_ production. The inhibitory neurotransmitter GABA inhibits this signaling cascade by Gαi-mediated inhibition of adenylyl cyclase activation, whereas the reduction in PGE2 formation caused by celecoxib additionally reduced adenylyl cyclase activation downstream of the Gαs-coupled PGE2 receptors. The resulting simultaneous inhibition of VEGF, CREB, MAPK, Src, and AKT pathways contributed to the significant inhibition of tumor progression.

Our data generated in vitro and in xenograft mouse models suggest, for the first time, that chronic exposure to epinephrine by psychological stress significantly stimulate the progression of PDAC via mechanisms primarily driven by COX-2 dependent arachidonic acid metabolites. Moreover, our in vitro identify both, cell proliferation and migration as mechanisms involved in stress-induced PDAC progression. Collectively, our findings suggest that the selective COX-2 inhibitor celecoxib may have strong inhibiting effects on the stress-induced progression of PDAC and that combination treatment of this agent with the inhibitory neurotransmitter GABA may significantly improve clinical outcomes in the absence and presence of psychological stress. These findings are in accord with previous reports that have described significant tumor-inhibiting effects of GABA treatment alone in pancreatic cancer cells in vitro [36] and in xenograft models [18]. The observed complete suppression of stress-induced xenograft progression by celecoxib indicates that the AA pathway plays a key role in stress-induced beta-adrenergic signaling (Fig. 8), an interpretation supported by the production of AA in response to beta-adrenergic agonists in pancreatic cancer cells [15].

Animal models that mimic the pattern of human disease play an important role in understanding the effects of stress on cancer and other diseases and in determining what inhibitors would be efficient to use to block tumor progression or further complications of disease. The consideration of chronic psychological stress caused by socio-economic factors and disease-related anxiety is an important and hitherto neglected aspect of cancer intervention that needs to be included in strategies for the prevention or therapy of pancreatic cancer to ensure success.

Stress is a complex process including environmental and psychosocial factors that initiate cascade information processing in both the peripheral and central nervous system [45]. Stress can be acute or chronic [46]. The catecholamine neurotransmitters noradrenaline and adrenaline are known to be elevated in individuals with acute or chronic stress [47]. Most stress-related effects of noradrenaline and adrenaline are mediated by β-ARs, which are the most extensively studied members of the G-protein-coupled receptor family [48]. β-ARs mediate many effects of catecholamines on target cells and have been identified in several cancer cell types, including pancreas [49], [15]. It has been shown that stress-induced increase in ovarian tumor growth was due to stimulation of tumor cell β-ARs, leading to elevated levels of VEGF that subsequently increase angiogenesis, thus illustrating multiple mechanisms by which β-AR stimulation of tumors can facilitate tumor growth [50]. In addition, it has been shown that chronic stress negatively impacts the outcomes of numerous diseases in men and women worldwide [18], [37], [47], [50] and that pancreatic cancer patients demonstrate particularly high levels of psychological stress [51]. In conjunction with our current findings, these observations suggest psychological stress as an important driving force in pancreatic cancer progression that can be successfully abolished by the targeted inhibition of stress-induced pathways.

We have shown that neurotransmitter responses to psychological stress significantly induced multiple signaling pathways that regulate the proliferation, migration, angiogenesis, and apoptosis of pancreatic cancer, resulting in a significant promotion of tumor growth in mouse xenografts [18]. In addition, our recent in vitro studies have identified an autocrine catecholamine loop in pancreatic cancer cells that activated the same pathways [38]. In conjunction with our current data, these findings warrant molecular profiling of pancreatic cancer to monitor the expression and activity levels of members of the regulatory cascade illustrated in Figure 8. Several of the β-adrenergic effectors (Fig. 8) that were strongly inhibited by celecoxib and GABA in our experiment are targets of pancreatic cancer therapy that have failed to improve clinical outcomes as single-agent treatments and are currently under investigation as combinations of multiple therapeutic agents [20], [21]. Our current findings suggest celecoxib treatment, accompanied by nutritional supplementation with GABA, as a promising new approach for pancreatic cancer intervention.

The overexpression of COX-2 results in the increased conversion of AA to prostaglandin, which then modulates multiple effectors as shown in Figure 8 and is in accord with reports on PGE2-induced angiogenesis [52] and metastasis [53]. It has been previously shown that the epidermal growth factor receptor (EGFR) and COX-2 pathways interact at several levels, and the EGFR is thought to be an important regulator of COX-2 expression via activation of the Ras/Raf/mitogen-activated protein kinase and activation of Akt and nuclear factor-kB pathways [54]. In addition, COX-2 reportedly mediates VEGVF and PGE_2_ production through increases in intracellular cAMP [55]. Our current findings identify stress-induced beta-adrenergic signaling as a potential regulator upstream of all of these effectors. Our data provide evidence that the stress-induced increase in pancreatic tumor growth was due to stimulation of tumor cell β-ARs and the associated stimulation of the AA pathway, EGF cascade, Src and AKT pathways, as well as VEGF interactions (Fig. 8). These multiple responses were significantly inhibited by celecoxib alone, effects significantly enhanced even further by additional treatment with GABA, a widely used nutritional supplement. Our current and recently published (18) data suggest that, irrespective of the expression of activating point mutations in K-ras, pancreatic cancer is highly sensitive to the cancer-stimulating effects of stress neurotransmitter driven responses to psychological stress. Our current data additionally indicate strong dependence of these stress responses on COX-2-dependent arachidonic acid metabolites, such as PGE_2_. These findings identify celecoxib as a promising agent for the prevention of stress-induced pancreatic cancer progression in clinical settings. The observed increase in the effectiveness of celecoxib by GABA identifies combination treatment with both agents during and after chemotherapy as a promising new approach to improve clinical outcomes of pancreatic cancer. This study has implications for cancer progression and prevention in populations under chronic psychological distress due to socio-economic and personal challenges, which are acerbated further when a person is diagnosed with a life-threatening disease such as pancreatic cancer.
